# Glucose-6-phosphate dehydrogenase deficiency allelic variants and their prevalence in malaria patients in Eritrea

**DOI:** 10.11604/pamj.2018.31.46.16527

**Published:** 2018-09-20

**Authors:** Yohannes Ghebremedhin Tseghereda, Joseph Kangethe Nganga, Andrew Nyerere Kimang’a, Tadesse Hagos Mehari, Yishak Gebrekidan Weldemichael

**Affiliations:** 1 Jomo Kenyatta University of Agriculture and Technology, Nairobi, Kenya; 2 National Commission for Higher Education, Asmara, Eritrea; 3 Eritrea Institute of Technology, Asmara, Eritrea

**Keywords:** G6PD deficiency, PCR-RFLP, Eritrea, zoba, ethnic groups, malaria

## Abstract

**Introduction:**

Glucose 6-phosphate dehydrogenase (G6PD) deficiency is the most common enzymopathy with a relatively high frequency in malaria-endemic regions. In Eritrea, there is scanty knowledge of G6PD deficiency. The aim of the study was to characterize and determine the prevalence of four common G6PD allelic variants.

**Methods:**

Three hundred and fourteen dried blood spot samples from unrelated microscopically diagnosed malaria patient Eritrean ethnic groups living in five zobas (regions) of Eritrea were analysed by PCR-RFLP method to identify the G6PD B, *G6PD A* (A376G), *G6PD A*-(G202A), and G6PD Mediterranean (C563T) variants. To confirm the RFLP results, samples positive for A376G but negative for G202A variants were subjected to Sanger sequencing and a subset of PCR products (exon 5) directly sequenced to identify A376G and other mutations.

**Results:**

For G6PD genotyping, *G6PD B* was detected in 87.5% and A376G detected in 12.5% of malaria patients, whereas G202A and C563T were absent. Bivariate Statistical analysis showed a statistically significant association between *G6PD* genotypes and zoba (P < 0.004 < 0.05). Sequencing revealed the expected A376G variant. In exon 5, four common (A376G) mutations, three uncommon mutations rs782669677 (535G→A) and one potentially new mutation (451G→C), relative to the reference, mRNA NM_001042351 were detected. Bioinformatic analysis of these mutations' potential functional impact suggests minimal effect on protein function.

**Conclusion:**

This is the first report indicating that *G6PD B* and *G6PD A* genotypes are prevalent in Eritrea. Similar findings were reported in neighboring countries. Further studies including phenotype analysis are needed to corroborate the observed results.

## Introduction

Malaria remains to be a public health concern with 429,000 reported associated deaths in 2015 in spite of the effort to control and eventually eliminate it has recorded huge success in the last decades worldwide [1]. Malaria is a public health concern in Eritrea although the country has scored tremendous achievements in its control over the past 15 years through intensive control interventions [2, 3]. Eritrea is located in the horn of Africa, where 67% of its populations live in malaria risk areas [4]. *Plasmodium falciparum* is responsible for 67% of malaria cases and *Plasmodium vivax* for 30%; there is a small percentage of cases due to mixed infections [5]. Eritrea has an estimated population of 5.19 million people [6] with nine officially recognized ethnic groups (Afar, Bilen, Hidareb, Kunama, Nara, Rashida, Saho, Tigre and Tigrigna). Tigrigna is the largest ethnic group followed by the Tigre group, whereas Rashaida is the smallest ethnic group in the country. Administratively, the country is divided into six zobas (regions): Maekel, Anseba, Gash Barka, Debub, Semienawi Keyih Bahri (Northern Red Sea) and Debubawi Keyih Bahri (Southern Red Sea). Among these zobas, Debub, Semienawi Keyih Bahri, Anseba and Gash Barka are endemic to malaria. Malaria in the country is mostly seasonal and unstable. The disease is transmitted through the *Anopheles arabiensis* primary vector, which is difficult to control using conventional interventions method due to its facultative indoor and/or outdoor feeding and resting behavior [7]. G6PD is a cytosolic enzyme present in all cells [8] and functions in the body's defense against oxidant damage [9]. In red blood cells (RBCs), G6PD is the only source of NADPH cofactor, which is needed for generating a reduced glutathione (GSH), the major antioxidant defense. *G6PD* gene encoding the G6PD enzyme is located on X chromosome and is highly polymorphic with more than 400 variants, described based on biochemical diagnosis [10]. G6PD deficiency is the most prevalent enzyme deficiency in humans [11] present in over 400 million people worldwide [12] with an estimated allelic frequency of 8% across malaria endemic countries [13]. As X-linked disorder, the transmission pattern results in G6PD deficiency hemizygous males or homozygous females, whereas heterozygous females can be either normal or deficient in G6PD activity because of mosaicism [14].

Although most individuals with G6PD deficiency are clinically asymptomatic, this defect causes neonatal jaundice, mild hemolytic anemia and chronic non-spherocytic hemolytic anemia triggered by infections, specific foods (fava beans), or drugs [15]. On the other hand, G6PD deficiency has protective power against malaria infection although whether this advantage is exclusive to heterozygous female individuals, or it is shared by both sexes equally has been controversial [16,17]. A case-control study performed on the coast of Kenya showed that only girls who were heterozygotes for the G6PD c.202T (G202A) variants were significantly protected from severe and complicated *Plasmodium falciparum* malaria [18]. Recent studies also revealed an increasing levels of G6PD deficiency associated with decreasing risk of cerebral malaria, but with increased risk of severe malarial anaemia [19]. To date, more than 400 G6PD variants have been identified, of which 186 variants are associated with G6PD deficiency by decreasing the activity or stability of G6PD [9, 20,21]. However, in sub-Saharan Africa three variants occur with polymorphic frequencies (> 0.1%): G6PD*B, G6PD*A and G6PD*A-. G6PD*B is the wild type and the most common variant in Africa and worldwide. G6PD*A has a single A→G substitution at nucleotide number 376. It is a normal variant with about 90% of the G6PD*B enzyme activity [22]. G6PD*A- is a deficient variant with about 8-20% of the wild type enzyme activity and caused by the A376G and G202A mutations [23]. G6PD Mediterranean variant is caused by the C563T mutation with less than 10% enzyme activity and found in Italy, Cyprus and in the Middle East [9]. In Eritrea, there is scant knowledge of G6PD deficiency. The Eritrean Government through its Ministry of Health is currently preparing pilot activities towards total elimination of malaria by 2030. Despite this, oxidative drugs and G6PD deficiency screenings are not part of the national policy for the treatment of *Plasmodium falciparum* and *Plasmodium vivax* infections in Eritrea. This study aimed at molecular characterization and determination of the prevalence of common *G6PD* variants, namely, *B, A, A-* and Mediterranean, in microscopically diagnosed malaria patient Eritrean ethnic groups.

## Methods

**Figure 1:**
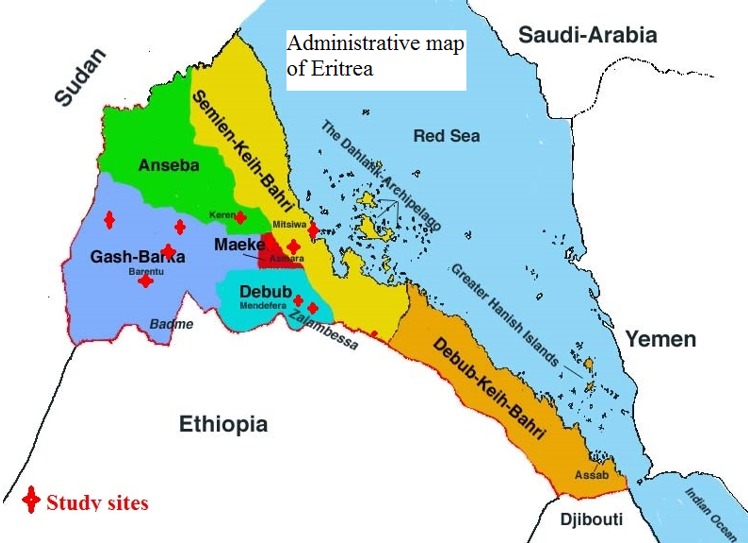
Map of Eritrea showing the ten study sites located in the selected five administrative zobas (regions) of Eritrea

**Study sites and study population:** this cross-sectional study was undertaken at 10 study sites located in five zobas of Eritrea (Figure 1) between August and October 2016. Study sites were selected based on practical accessibility, on the availability of malaria cases and geography. The number of study participants enrolled from each study site was determined using probability proportional to size (PPS). To limit double sampling of genetically related individuals, a single individual per family was recruited. Individuals born from parents of two different ethnic groups were excluded from this study.

**Microscopic examination of malaria parasite:** outpatients visiting health stations/centres/hospitals and those clinically suspected to have malaria were diagnosed for *Plasmodium* species by microscopy. Thick and thin blood smears and dried filter paper blood spots (DBS) were made from a drop of finger-prick blood. After the slides were air-dried, the thin smear fixed in methanol and both smears were stained with 10% Giemsa for 15 minutes. The stained slides were subsequently air-dried and viewed under 100X oil immersion microscope (Olympus microscope CX21, Tokyo, Japan). An experienced microscopist read the slides. Species identification was done by visualizing thin blood smear. Parasitaemia was determined as the percent of malaria parasites observed per 200 white blood cells [24].

**Dried blood spot sample collection for genetic analysis:** three hundred and fourteen finger-prick dried blood spot (DBS) samples were collected on Whatman 903^TM^ filter paper cards from microscopically diagnosed malaria patients (136 females and 178 males) Eritrean ethnic groups. None of the samples contain any personnel identifiers. Therefore, DBS samples were collected from Tigrigna (n=145), from Tigre (n=67), from Nara (n=35), from Saho (n=23), from Hidareb (n=15), from Kunama (n=14), from Bilen (n=14), from Rashaida (n=1) ethnic groups. Notably Tigrigna is the largest group followed by Tigre, while Rashaida is the smallest ethnic group in Eritrea. After properly air-dried at room temperature and individually packaged in a small zip-lock plastic bag with two desiccant bags, DBS samples were stored at 4°C during the fieldwork, and then transported to the National Health Laboratory (NHL), in Asmara, Eritrea for storage at -20°C. Subsequently all DBS samples were transported to the Laboratory of Molecular Anthropology in the Department of Biological, Environmental and Geological Sciences (BiGeA), University of Bologna, Italy for genetic analysis.

**Ethical consideration:** ethical approval for this study was obtained from the Health Research Proposal Review and Ethical Clearance Committee of Eritrea and the Ministry of Local Government of all six zobas of Eritrea. Informed consent was obtained from all participants or from parents for children aged less than 15 years. All molecular laboratory activities were performed with permission from the Department of BiGeA, University of Bologna, Italy.

**DNA extraction and genotyping:** three punched out circles were placed in a 1.5 ml microcentrifuge tube and DNA was extracted using QIAamp DNA blood Mini Kit (Qiagen, Germany) according to the manufacturer's recommendation for dried blood spots. DNA was eluted in 100 μL of elution buffer and used as templates to search for the more common *G6PD* genetic variants whose mutations are located within the exons, 3 + 4, 5 and 6 of the *G6PD* gene. A polymerase chain reaction (PCR) using three primer pairs as described by Tishkoff *et al*. [25] to amplify the corresponding regions of exons 3+4-6 in a Biometra TGradient PCR machine. PCR reactions were performed in 25 μL volumes containing 6 µL of genomic DNA, 5X Green GoTaq^®^ Flexi Buffer, 10 mM of each dNTP, 10 μM of each primer, 25 mm of MgCI_2_, and 1.25 units of GoTag DNA polymerase using the Promega GoTaq^®^ Flexi DNA Polymerase Kit (Madison, WI). Genomic DNA was first amplified exon 5 for A376G: G6PD5F (5'-CAAAGAGAGGGGCTGACATC-3'and G6PD5R (5'-CTCATAGAGTGGTGG GAGC-3'). PCR thermo-cycling condition was performed as follows: initial denaturation at 94°C for 2 min, followed by 34 cycles of denaturation at 94°C for 1 min, annealing at 60°C for 1 min and extension at 72°C for 30 sec and a final extension step at 72 °C for 5 min. PCR products were analyzed by 1% agarose gel electrophoresis and midori green dye staining, and cleaved to perform the restriction fragment length polymorphism (RFLP) analysis with 10 units of *FokI*, *NlaIII*, and *MboII* endonucleases under conditions recommended by the manufacturer (New England BioLab Inc, USA). Digestions were performed in 96 well microwell plates in a final volume of 35.5 µl. PCR products were incubated at 37°C for 1 hour with *FokI* to identify the *G6PD A* (A376G) variant. All samples showing positive for A376G were further amplified using primers for G202A: G6PD3/4F (5'-AACCACACACCTGTTCCCTC-3') and G6PD3/4R (5'-GCTGGTAGAGAGG GCAGAAC. Amplicons were incubated at 37°C for 50 min with *NlaIII* to identify the G6PD A- (G202A) variant. DNA was amplified using primers, G6PD6F (5'-TGCAGCTGTGATCCTCACTC-3' and G6PD6R (5'-AGGTGGAGGAACTGACCTTG-3'). PCR products were then incubated at 37°C for 40 minutes with *MboII* to identify the G6PD Mediterranean (C563T) variant. All RFLP products were separated by 3% nusieve-agarose gels stained with midori green dye at occurrence of 100 bp DNA size marker. Gels were visualized using UV light and sex was determined by the number of bands (three for females and two for males).

**DNA sequencing:** to confirm RFLP analysis results, samples showing positive for A375G, but negative for G202A variants were further analyzed by DNA sequencing in one direction [26]. A subset of PCR products of exon 5 of the G6PD gene was also directly sequenced to identify A376G and other mutations. PCR products of G6PD exons, 3+4 and 5 were amplified using forward primers previously described in Tishkoff *et al.* [25]. PCR thermal cycling protocol was performed as previously described in Promega Madison, USA. PCR products were purified using ExoSap enzymes (USB, Affymetrix, USA) and subjected to cycle sequencing using Big Dye Terminator version 1.1 Cycle Sequencing Kit (Applied Biosystems) according to the manufacturer's instructions. After cycle sequencing, sequenced products were purified using ethanol precipitation and re-suspended in 10 µL injection solution. Purified cycle sequencing products then subjected to capillary electrophoresis (Applied Biosystems) in an ABI PRISM 3730 DNA genetic analyzer (Applied Biosystems) at the Operative Unit of Medical Genetics of Bologna Hospital, Italy. Substitution of nucleotides were identified using the wild type GenBank reference sequence (GRCh38: X: 154535096:154535437:1).

**Phylogenetic analysis:** phylogenetic analysis was performed using the nucleotide sequences obtained from G6PD exon 5 PCR products. The nucleotide sequences were first manually edited by removing ambiguous sequences at the ends and generating reverse complements, which were then aligned using a Geneious version 11.1 [27]. Human *G6PD exon* 5 sequence was extracted from NM_001042351.2 and GRC38/hg38 reference sequences in the NCBI nucleotide and human genome databases were used as wildtype allele B controls. Sample sequences with more than two mismatches to wildtype were assumed to contain sequence errors whereas sample sequences with only one or two mismatches to wildtype were assumed possibly to represent genuine variants. Multiple sequence alignments by Clustal Omega version 1.2.4 were generated using a previously identified uncommon Ethiopian G6PD variant (rs782669677) from West Ethiopia [28] to highlight the correspondence between them.

**Data analysis:** data was entered in Microsoft Excel sheet and transferred to SPSS version 23.0 (IBM Statistic data, USA) for statistical analysis. Descriptive statistics such as proportions was used to summarize categorical v ariables. Bivariate logistic regression analysis was used to measure the strength of association between G6PD genotypes and demographic data. A P value <0.05 was considered statistically significant.

## Results

To characterize and determine the prevalence of common G6PD variants among eight Eritrean ethnic groups, finger prick dried blood spot (DBS) samples were obtained from 314 microscopically diagnosed malaria patients (136 females and 178 males) residing in five zobas (regions) of Eritrea. The microscopic examination revealed 284 *P. falciparum* infections, 28 *P. vivax*, and two *P. falciparum* and *P. vivax* mixed infection (Table 1). The median age of study participants was 28 years, and 75% of the study subjects were under 42 years.

**Table 1:** Malaria parasite species detected by microscopy according to demographic characteristics

Category	*P. falciparum*		*P.vivax*		Mixed infection*		Total
	n	%	n	%	n	%	n
**Sex**							
Female	124	89.9	14	10.1	0	0.0	138
Male	160	90.9	14	8.0	2	1.1	176
**Ethnicity**							
Bilen	11	78.6	2	14.3	1	7.1	14
Hidareb	15	100	0	0.0	0	0.0	15
Kunama	14	100	0	0.0	0	0.0	14
Nara	32	94.1	2	5.9	0	0.0	34
Rashaida	1	100	0	0.0	0	0.0	1
Saho	22	95.7	1	4.5	0	0.0	23
Tigre	62	91.2	5	7.4	1	1.5	68
Tigrigna	127	87.6	18	12.4	0	0.0	145
**Age groups**							
⋅10	43	93.50	3	6.5	0	0.0	46
10-19	92	92.00	7	7.0	1	1.0	100
20-29	54	90.0	6	10	0	0.0	60
30-39	38	90.5	4	9.5	0	0.0	42
40-49	20	83.3	3	12.5	1	4,2	24
⋅ 50	37	88.1	5	11.9	0	0.0	42
**Zobas (Regions)**							
Maekel	13	61.9	8	38.1	0	0.0	21
Anseba	21	72.4	7	24.1	1	3.4	29
Gash Barka	139	93.3	9	6.1	0	0.0	148
Debub	46	95.8	2	4.2	0	0.0	48
Semienawi Keyih Bahri	65	95.6	2	2.9	1	1.5	68
Maekel	13	61.9	8	38.1	0	0.0	21
Anseba	21	72.4	7	24.1	1	3.4	29

* = P.falciparum + P.vivax

**Molecular findings and prevalence of G6PD variants:** the PCR-RFLP analysis showed that 272 (87.5%) out of 311 clinically determined malaria patients carried the normal G6PD allele and 39 (12.5%) the *G6PD A*(A376G) variant. No *G6PD A-* and *G6PD* Mediterranean variants were found. African G6PD variants were confirmed by DNA sequencing. Notably, those individuals who lacked both *FokI* and *NIaIII* restriction sites were classified as B [25]. Of the 12.5% genotyped malaria patients with *G6PD B* genotype, 88.1% (119/135) were females and 86.9% (153/176) were males, and no significant association between *G6PD B* and sex was observed (P = 0.748, bivariate logistic regression analysis). Overall prevalence of G6PD deficiency was 12.5% (39/311) in malaria patients. Of the 39 genotyped malaria patients with *G6PD A* variant, 15 (4.8%) were A heterozygous females (type AB), 1 (0.3%) was an A homozygous female (type AA), and 23(7.4%) were A hemizygous males (type A). The highest proportion of *G6PD A* variant was observed in males (13.1%) than in females (11.9%). Bivariate logistic regression analysis revealed no statistically significant difference between G6PD A variant and sex (P > 0.05). The prevalence of the *G6PD A* variant frequency with respect to ethnic groups was recorded: 14.3% in Bilen, 0% in Hidareb, 21.4% in Kunama, 2.9% in Nara, 8.7% in Saho, 7.4% in Rashaida/Tigre and 18.3% in Tigrigna. Highest prevalence was showed in malaria patients of Nara ethnic group with a frequency of 21.4%. In contrast, no A376G variant was observed in malaria patients in Hidareb ethnic group. For the validity analysis, the study has also grouped the ethnic groups based on their ethno-linguistic origins and analyzed. It was observed that the prevalence of A376G variant was 7.7% in Cushitic, 8.2% in Nilotic and 14.8% in Semitic; No statistically significant differences were assessed between G6PD genotypes and ethnic groups and ethno-linguistic origins (P > 0.05). With regards to zobas, the prevalence *G6PD A* (A376G) variant frequency was 8.3% in Maekel, 10.0% in Anseba, 12.4% in Gash Barka, 28.3% in Debub, and 3.6% in Semienawi Keyih Bahri. The highest frequency of A376G variant was observed in individuals residing in zoba Debub (28.3%), and a statistically significant difference of A376G variant and zoba was observed (P < 0.004 < 0.05). The distribution of *G6PD B* and *G6PD A* (A376G) with respect to demographic data in malaria patient Eritrean ethnic groups is summarized in Table 2. In this study, *G6PD A-* (G202A) and Mediterranean (C563T) variants were also successfully genotyped in the analyzed population. Of the 311 genotyped malaria patients, 0.00% (0/311) carried the G202A and C563T variants.

**Table 2:** Distribution of G6PD allelic variants by ethnic groups, ethno-linguistic groups and zobas (regions)

categories	Total	G6PD genotypes				
		A				B
		Heterozygous female	Homozygous female	Hemizygous male	Total n(%)	Total n(%)
**Ethnic gropus**						
Bilen	14	0	0	2	2(14.3)	12(85.7)
Hidareb	15	0	0	0	0,0	15(100)
Kunama	14	2	0	1	3(21.4)	11(78.6)
Nara	35	0	1	0	1(2.9)	34(97.1)
Saho	23		0	2	2(8.7)	2(8.7)
Tigre/Rashida	68	3	0	2	5(7.4)	63(92.6)
Tigrigna	142	10	0	16	26 (18.3)	116(81.7)
**Ethno-linguistic groups**						
Cushitic	52	0	0	4	4(7.7)	48(92.3)
Nilotic	49	2	1	1	4(8.2)	45(91.8)
Semitic	210	13	0	18	31 (12.85)	179(87.15)
**Zobas**						
Maekel	20	0	0	0	2(10.0)	18(90.0)
Anseba	46	4	0	9	13 (28.3)	33(71.7)
Debub	153	8	1	10	19 (12.4)	134(87.6)
Gash Barka	36	1	0	2	3(8.3)	33(91.7)
Semienawi Keyih Bahri	56	2	0	0	2(3.6)	54(96.4)

**DNA sequencing results:** thirty one out of 39 sequenced products of exons, 3+4 had the expected A376G mutation (T183C with respect to the PCR fragment) and eight samples were unreadable. Of the 68 out of 70 sequenced products of exon 5, 3/68 (4.41%) of individuals carried common A376G mutations (rs1050829, 376 A→G, chrX: 154535277; 156 asn → asp) and 3/68 (4.41%) of individuals had uncommon mutations (rs782669677, 535 G→A, chrX: 154535208; 179: ala→thr). The mutation 535G→A results in an Alanine→Threonine amino acid change at position 149 in protein isoform B (Accession No NP_001035810.1) from mRNA v. 2 (Accession No NM_001042351.2). And 1/68 (1.47%) of individual had a novel mutation (G451C; mRNA NM_00104235).

## Discussion

This is the first study performed to genotype and determine the prevalence of four common G6PD variants by PCR-RFLP in microscopically confirmed malaria patients living in five zobas of Eritrea. It was found that the *G6PD* B and G6PD A (A376G) variants are quite prevalent in individuals with predominantly *P. falciparum* and *P. vivax* infections. The work enabled us to understand the genetic basis of G6PD deficiency in Eritrean populations. Eritrea is a multilingual and multiethnic nation. Its various ethnic groups are unequally distributed throughout the country. The prevalence of the normal *G6PD B* allele was 87.5% (272/311) among the malaria patients, which is in line with the literature that the wild type *G6PD B* allele is the most common variant in Africa and worldwide [22]. Similarly, other authors found a high prevalence of *G6PD B* genotype in Burkina Faso (74.5%) [11] and reported 53.6-81.5% among inhabitants of sub-Saharan Africa (Sierra Leone, Mende, Temne, Ghana, Fante, Ga, Cameroon, and Bakaka) [25]. The overall prevalence of *G6PD A* variant was 12.5% observed in the analyzed microscopically diagnosed malaria patients. Of the 39 (12.5%) genotyped individuals with A376G variant, 4.8% were heterozygous females , 0.32% was a homozygous female, and 7.4% were homozygous males for A376G variant, which is in agreement with previously reported results from Ethiopia, of which 23.26% (20/86) genotyped malaria patients with A376G variant, 19 were heterozygous females and one hemizygous male carried the A376G mutation [28]. The prevalence of *G6PD A* variant by sex was 11.9% in females and 13.1% in males in this study, but these difference are not statistically significant (P > 0.05). The higher prevalence of G6PD deficiency in male is consistent with other studies conducted in Afghanistan [29] and Solomon Islands [30]. The male predominance of G6PD deficiency can be attributed to the X-linked inheritance in G6PD gene [9]. The prevalence of the A376G variant in terms of frequency among the ethnic groups ranged from 0.00% to 21.4%. Similar results were reported from a study done by G6PD fluorescent spot test among unrelated males of Karen (4.1%) and Burman (12.9%) ethnic groups in a malaria endemic region on the Thailand-Myanmar border [31] and in five ethnic groups in Pakistan [32]. In this study, higher *G6PD A* (A376G) variant prevalence was found to be associated with individuals residing in zoba Debub (28.3%) followed by zoba Gash Barka (12.4%) and these differences were statistically significant (P < 0.004 < 0.05) which is in agreement with previous findings in Ethiopia and Nepal [33, 34]. Notably, the 8.3% of malaria patients with the A376G variant recorded in zoba Maekel, which is considered malaria free zone of Eritrea. These individuals who are mainly Tigrigna and Tigre ethnic groups resided and/or temporarily travelled to different zobas, and they had come for their medical treatment. The frequency of *G6PD* did differ by zoba (region) due to differing ethnic make-up of each zoba. As conclusion, the frequency of G6PD deficiency differed across the world depending on the ethnic groups and regions [13-15, 35].

Considerable evidences showed that in African populations the G6PD A- (G202A/A376G) mutations is exclusively the cause of G6PD deficiency. Few studies reported *G6PD A-* (202GA) frequency estimates considerably lower than those generally found in sub-Saharan Africa [10] and confirmed by a recent geostatistical model-based map that predicted a 1.0% prevalence [15]. The prevalence G202A deficient variant was zero (0.0%) in this study, which is in line with the study findings in Ethiopia [28, 36] and Northern Sudan [37]. In contrast, the frequency of *G6PD A-* deficient variant was higher in Uganda (20.41%) and in Mali (7.9%) respectively [38, 39]. The absence of *G6PD A-* mutation in this study could be due to geographical location and the molecular heterogeneity of G6PD deficiency among different populations [40]. G6PD Mediterranean variant is primarily found in populations within the Mediterranean region [41, 42]. The prevalence of the *G6PD* Mediterranean (C563T) mutation in terms of frequency was zero (0.00%) in this study, which is in accordance with studies done in Angola [43] and in Ethiopia [28]. In contrast, Mediterranean mutation is the second most common variant in some African Arab countries like Algeria and Tunisia with frequencies of 23% and 11.4%, respectively [44]. This mutation probably arose in the Mediterranean basin and spread to the Middle East and North Africa through extensive trade routes and colonization by the Greeks. The distribution pattern of the Mediterranean mutation is consistent with spread through the army of Alexander the Great who occupied the Middle East and North Africa and went as far east as India [45]. The absence of Mediterranean C563T mutation in this study could be due to the absence of gene flow from Mediterranean regions and North Africa in addition to the non-occurrence of this particular mutation among the current study participants. Sequencing confirmed the presence of common (A376G) mutation. Additionally, the prevalence of common mutation (A376) was 4.41% (3/68) and uncommon mutation (G535A) was 4.41% (3/68) among the malaria patients in exon 5 of the *G6PD gene*. In contrast, among 86 sequenced malarial samples from Ethiopia there were 20/86 (23%) common A376G mutations, and 2/86 (2.3%) uncommon mutations (rs782669677, G535A in heterozygous females. Therefore, compared to Ethiopia, malaria patients Eritrean population in this case have a much lower prevalence of the common A376G variant, but a higher prevalence of the rarer G451C mutation. Bioinformatic analysis of these mutations' suggests that they have minimal effect on G6PD protein function [28].

## Conclusion

This study is the first report on molecular characterization and the prevalence of G6PD allelic variants in microscopically confirmed malaria patients in Eritrea. PCR-RFLP results and DNA sequencing showed the prevalence of non-deficient *G6PD B* and *G6PD A* (A376G) variants while the G202A and C563T variants were absent in this study. The discovery of uncommon mutations one potentially novel mutation gives the impetus for further investigation into *G6PD* variation in Eritrea. Our findings can contribute in filling the knowledge gap to the overall molecular characterization and prevalence of G6PD genotypes in Eritrea and in world populations. The presented data suggest the use of primaquine treatment without considerable safety concerns in Eritrea. Further studies including phenotypic approaches are needed to confirm the observed results. Some limitations exist in the study. The sample size of certain ethnic groups was very small in this study, thus more data is required from each ethnic groups to accurately estimate the prevalence of G6PD genotypes in Eritrean population. There was lack of positive internal quality control material for G6PD deficient C563T variant and was not confirmed by sequencing due to fund constraint.

### What is known about this topic

- Glucose-6-phosphate dehydrogenase (G6PD) deficiency is X-linked hereditary defect affects over 400 million people particularly males;
- G6PD has over 186 mutations responsible for G6PD deficiency;
- G6PD deficiency is the most common in malaria endemic areas and has a protective power against malaria.

### What this study adds

- This study is the first of its kind carried out to characterize and determine prevalence of common G6PD variants in Eritrea;
- Assessed the distribution of G6PD variants among the ethnic groups;
- The outcome of this study shed light on appropriateness of current existing malaria treatment in Eritrea.

## Competing interests

The authors declare no competing interest.
